# Convergent Evolution of HLA-C Downmodulation in HIV-1 and HIV-2

**DOI:** 10.1128/mBio.00782-20

**Published:** 2020-07-14

**Authors:** Kristina Hopfensperger, Jonathan Richard, Christina M. Stürzel, Frederic Bibollet-Ruche, Richard Apps, Marie Leoz, Jean-Christophe Plantier, Beatrice H. Hahn, Andrés Finzi, Frank Kirchhoff, Daniel Sauter

**Affiliations:** a Institute of Molecular Virology, Ulm University Medical Center, Ulm, Germany; b Centre de Recherche du CHUM, Montreal, Canada; c Département de Microbiologie, Infectiologie et Immunologie, Université de Montréal, Montreal, Canada; d Department of Medicine, Perelman School of Medicine, University of Pennsylvania, Philadelphia, Pennsylvania, USA; e NIH Center for Human Immunology, National Institutes of Health, Bethesda, Maryland, USA; f Normandie Université, UNIROUEN, UNICAEN, GRAM 2.0, Rouen, France; g Normandie Université, UNIROUEN, UNICAEN, GRAM 2.0, Rouen University Hospital, Department of Virology, Laboratory Associated with the National Reference Center on HIV, Rouen, France; h Department of Microbiology and Immunology, McGill University, Montreal, Canada; Simon Fraser University; University of Pittsburgh School of Medicine

**Keywords:** HIV-1, HIV-2, SIV, HLA-C, Vpu, Vif

## Abstract

HLA-C-mediated antigen presentation induces the killing of human immunodeficiency virus (HIV)-infected CD4^+^ T cells by cytotoxic T lymphocytes (CTLs). To evade killing, many HIV-1 group M strains decrease HLA-C surface levels using their accessory protein Vpu. However, some HIV-1 group M isolates lack this activity, possibly to prevent the activation of natural killer (NK) cells. Analyzing diverse primate lentiviruses, we found that Vpu-mediated HLA-C downregulation is not limited to pandemic group M but is also found in HIV-1 groups O and P as well as several simian immunodeficiency viruses (SIVs). We show that Vpu targets HLA-C primarily at the protein level, independently of its ability to suppress NF-κB-driven gene expression, and that in some viral lineages, HLA-C downregulation may come at the cost of efficient counteraction of the restriction factor tetherin. Remarkably, HIV-2, which does not carry a *vpu* gene, uses its accessory protein Vif to decrease HLA-C surface expression. This Vif activity requires intact binding sites for the Cullin5/Elongin ubiquitin ligase complex but is separable from its ability to counteract APOBEC3G. Similar to HIV-1 Vpu, the degree of HIV-2 Vif-mediated HLA-C downregulation varies considerably among different virus isolates. In agreement with opposing selection pressures *in vivo*, we show that the reduction of HLA-C surface levels by HIV-2 Vif is accompanied by increased NK cell-mediated killing. In summary, our results highlight the complex role of HLA-C in lentiviral infections and demonstrate that HIV-1 and HIV-2 have evolved at least two independent mechanisms to decrease HLA-C levels on infected cells.

## Abstract (precis)

Genome-wide association studies suggest that HLA-C expression is a major determinant of viral load set points and CD4^+^ T cell counts in HIV-infected individuals. On the one hand, efficient HLA-C expression enables the killing of infected cells by cytotoxic T lymphocytes (CTLs). On the other hand, HLA-C sends inhibitory signals to natural killer (NK) cells and enhances the infectivity of newly produced HIV particles. HIV-1 group M viruses modulate HLA-C expression using the accessory protein Vpu, possibly to balance CTL- and NK cell-mediated immune responses. Here, we show that the second human immunodeficiency virus, HIV-2, can use its accessory protein Vif to evade HLA-C-mediated restriction. Furthermore, our mutational analyses provide insights into the underlying molecular mechanisms. In summary, our results reveal how the two human AIDS viruses modulate HLA-C, a key component of the antiviral immune response.

## INTRODUCTION

HLA-A, -B, and -C represent the three classical major histocompatibility complex class I (MHC-I) proteins in humans and are well known for their ability to present cellular and foreign peptides to cytotoxic T lymphocytes (CTLs). To evade CTL-mediated killing, human immunodeficiency virus type 1 (HIV-1), HIV-2, as well as simian immunodeficiency viruses (SIVs) decrease HLA-A and -B protein levels on infected cells using their accessory protein Nef (1). More recently, it has become clear that many HIV-1 group M strains use their accessory protein Vpu to also downmodulate HLA-C (2–6). Although the original report of HLA-C downregulation by HIV showed that an HIV-2 strain could also downregulate HLA-C (2), how HLA-C was modulated by HIV-2, which lacks Vpu, and other primate lentiviruses remained unclear.

Compared to the highly polymorphic HLA-A and -B alleles, HLA-C shows less variation and is expressed at about 13- to 18-fold-lower levels (7). Nevertheless, HLA-C plays a key role during HIV-1 replication, and genome-wide studies identified HLA-C as a main determinant of viral loads and CD4^+^ T cell depletion in HIV-1-infected individuals (8, 9). Interestingly, HLA-C plays opposing roles during HIV-1 replication. On the one hand, HLA-C promotes CTL-mediated killing of HIV-1-infected cells by presenting virus-derived peptides (10–14), including those that are only poorly presented by HLA-A and HLA-B (15, 16). Consistent with this, HLA-C-mediated CTL pressures select for rapid viral escape (17–19). On the other hand, HLA-C interacts with a variety of inhibitory receptors on natural killer (NK) cells (6, 20–22). This is particularly important in the context of Nef-mediated downmodulation of HLA-A and -B, where residual HLA-C prevents a process termed “missing-self detection,” in which NK cells detect and kill cells without MHC-I molecules on their surface (23). Furthermore, HLA-C is believed to be incorporated into budding virions and to enhance HIV-1 particle infectivity, possibly by modulating the conformation of the viral envelope glycoprotein (Env) (24–28). Despite this dual role of HLA-C during HIV-1 replication, HLA-C protein levels on peripheral blood CD3^+^ cells correlate inversely with HIV-1 loads (29, 30), suggesting that its antiviral effects usually prevail.

Although HLA-C is downmodulated by HIV-1 group M Vpu (2–5), this accessory protein exerts multiple functions, which can vary among different viral lineages. Notably, the markedly different spread of HIV-1 groups M, N, O, and P in the human population appears to be associated with varying Vpu activities (31). Specifically, the Vpu protein of pandemic group M viruses promotes the release of budding virions by efficiently counteracting the host restriction factor tetherin, while the Vpu proteins of group O and P viruses lack this activity, and the Vpu protein of group N viruses is only poorly active (32–34). Furthermore, the Vpu proteins of group N viruses lost their ability to downmodulate CD4, the main entry receptor of HIV, from the surface of infected cells, an activity that is conserved among the Vpu proteins of group M, O, and P viruses (32, 33). To investigate whether the downmodulation of HLA-C is another variable function, we analyzed a set of 33 Vpu proteins representing all four groups of HIV-1, SIVcpz from chimpanzees, as well as SIVs infecting *Cercopithecus* monkey species. We found that Vpu-mediated HLA-C downmodulation is not limited to HIV-1 group M but also is present in HIV-1 group O, HIV-1 group P, as well as several SIV lineages. Remarkably, we also found that HIV-2, which lacks a *vpu* gene, has evolved the ability to decrease HLA-C surface expression using its accessory protein Vif. Consistent with the different Vpu-mediated effects on CTL- and NK cell-mediated killing, we demonstrate that HLA-C downmodulation by HIV-2 Vif also coincides with increased killing of infected T cells by NK cells. Together with mutational analyses and inhibitor studies, these findings provide mechanistic insights into how primate lentiviruses evade cell-mediated antiviral immune responses and identify a fascinating example of convergent evolution.

## RESULTS

### HIV-1 groups M, O, and P as well as related SIVs use Vpu to downmodulate HLA-C.

**FIG 1:**
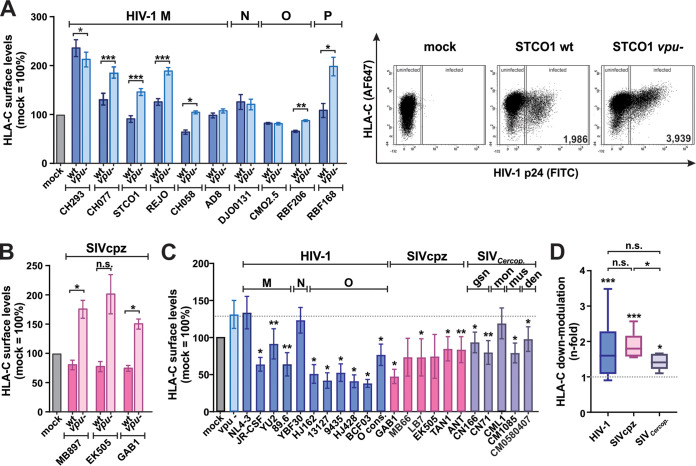
Vpu-mediated HLA-C downmodulation in HIV-1 and related simian immunodeficiency viruses. (A to C) Purified CD4^+^ T cells were infected with the indicated wild-type (wt) or *vpu*-defective (*vpu−*) clones representing HIV-1 groups M to P (A) and SIVcpz (B) or HIV-1 NL4-3 chimeras expressing the indicated heterologous *vpu* genes (C). At 3 days postinfection, HLA-C surface levels in infected cells were determined by flow cytometry and normalized to the value for uninfected control cells. Representative primary data are shown on the right in panel A. Bar diagrams show mean values from three to eight independent experiments ± standard errors of the means (SEM). AF647, Alexa Fluor 647. (D) Average *n*-fold downmodulation of HLA-C by Vpu proteins of HIV-1, SIVcpz, or SIVs isolated from *Cercopithecus* species (SIV*_Cercop_*_._). Values are derived from the experiments shown in panels A to C. Box-and-whisker-plots illustrating the minimum, maximum, sample median, and first and third quartiles are shown. Asterisks indicate statistically significant differences between *vpu*^+^ and *vpu*-defective samples (*, *P* < 0.05; **, *P* < 0.01; ***, *P* < 0.001; n.s., not significant).

To investigate the conservation of Vpu-mediated HLA-C downmodulation in different groups of HIV-1, we performed flow cytometric analyses of purified human primary CD4^+^ T cells infected with wild-type (wt) or *vpu*-deficient infectious molecular clones (IMCs) of HIV-1 groups M, N, O, and P. In agreement with previous studies analyzing group M viruses (2, 4, 5), the ability of Vpu to decrease HLA-C levels varied among different clones of HIV-1 (Fig. 1A). While Vpus of most HIV-1 strains, including members of groups M (CH077, STCO1, REJO, and CH058) O (RBF206), and P (RBF168), reduced HLA-C cell surface levels by up to 45%, some had no significant effect on HLA-C expression. The presence of this Vpu activity in different groups of HIV-1 suggests that it evolved in their ape precursors, i.e., before the emergence of HIV-1/AIDS in the human population. Indeed, Vpus of SIVcpz strains reduced HLA-C levels by 50 to 61% (Fig. 1B).

Vpu is encoded not only by HIV-1 and SIVcpz but also by SIVs infecting guenon monkeys (35). As molecular clones are not available for most of these viruses, we analyzed the activity of the respective Vpu proteins when expressed from an HIV-1 NL4-3 backbone (32). Again, all HIV-1 (except for a second strain of group N and the cell culture-adapted strain NL4-3) and all SIVcpz Vpus significantly decreased HLA-C levels on infected primary cells (Fig. 1C). Furthermore, Vpus from SIVs infecting greater spot-nosed (SIVgsn), mustached (SIVmus), and Dent’s (SIVden) monkeys also reduced HLA-C surface levels by 26 to 40%. In summary, our results show that Vpu-mediated HLA-C downmodulation is not only a characteristic of pandemic HIV-1 group M but also found in the less prevalent HIV-1 groups O and P as well as related SIVs infecting various primate species (Fig. 1D).

### Vpu downmodulates HLA-C independently of its ability to inhibit NF-κB activity.

**FIG 2:**
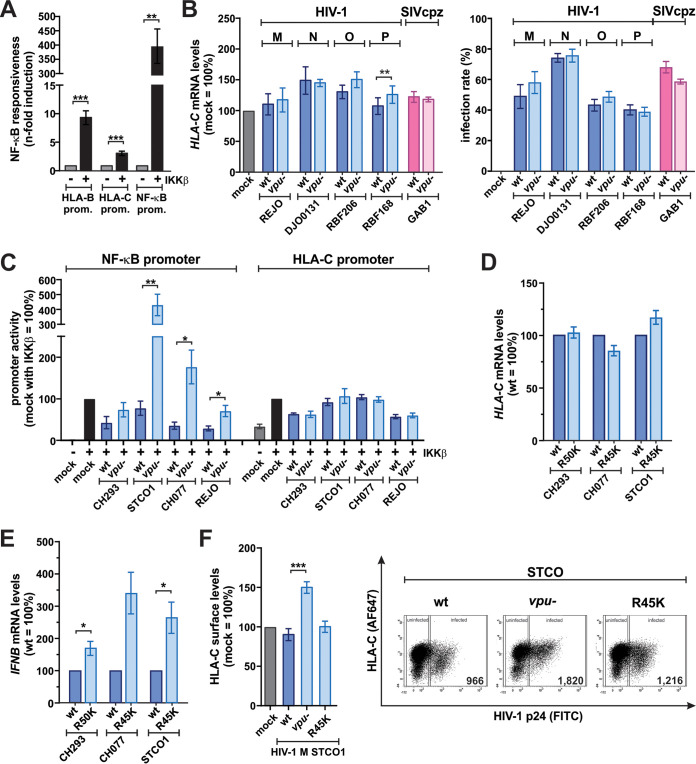
HLA-C downmodulation and NF-κB inhibition are separable functions of Vpu. (A) HEK293T cells were transfected with reporter constructs expressing firefly luciferase under the control of the HLA-B or HLA-C promoter or an artificial promoter harboring three NF-κB binding sites. A control vector expressing *Gaussia* luciferase under the control of a minimal promoter was cotransfected for normalization, and NF-κB was activated by transfection of a constitutively active mutant of IKKβ. At 2 days posttransfection, luciferase activities were determined, and NF-κB responsiveness was calculated. (B) Purified CD4^+^ T cells were infected with the indicated wild-type (wt) or *vpu*-defective (*vpu−*) viruses. At 3 days postinfection, *HLA-C* mRNA levels (left) and infection rates (right) were determined by qRT-PCR and flow cytometry, respectively. (C) HEK293T cells were transfected and promoter activity was determined as described above for panel A. Proviral constructs were cotransfected as indicated. (D and E) Purified CD4^+^ T cells were infected with the indicated HIV-1 clones. R45K and R50K describe point mutations in Vpu that selectively abrogate its ability to inhibit NF-κB signaling (39). At 3 days postinfection, *HLA-C* (D) and *IFNB* (E) mRNA levels were determined by qRT-PCR. (F) Purified CD4^+^ T cells were infected with the indicated variants of STCO1, and HLA-C protein levels at the cell surface were determined by flow cytometry as described in the legend of Fig. 1. Representative primary data are shown on the right. Bar diagrams show the mean values from three to eight independent experiments ± SEM. Asterisks indicate statistically significant differences (*, *P* < 0.05; **, *P* < 0.01; ***, *P* < 0.001).

Vpus from diverse primate lentiviruses, including those of SIVgsn and SIVmus, efficiently suppress the activation of NF-κB (36–41). As a consequence, the expression of NF-κB target genes such as HLA-B is reduced (41). Since the HLA-C promoter harbors a putative NF-κB binding site (42), we hypothesized that Vpu may not only target HLA-C at the protein level (3) but also suppress its mRNA expression. In agreement with this, we found that canonical NF-κB signaling activates the promoters of both HLA-B and, to a lesser extent, HLA-C (Fig. 2A). However, quantitative reverse transcription-PCR (qRT-PCR) analyses showed that a representative subset of HIV-1 and SIV Vpus did not markedly alter *HLA-C* mRNA levels in infected primary human CD4^+^ T cells (Fig. 2B), although they reduced HLA-C surface levels (Fig. 1) and inhibited NF-κB signaling (41). Similarly, Vpu suppressed the activation of a consensus NF-κB promoter but had no effect on HLA-C promoter-driven gene expression (Fig. 2C). Furthermore, the previously described R45K and R50K mutations known to selectively abrogate the ability of Vpu to inhibit NF-κB (39) had no significant effect on *HLA-C* mRNA levels (Fig. 2D) but resulted in increased NF-κB-driven expression of the *IFNB* gene (Fig. 2E). Consistent with this, the R45K mutant of HIV-1 STCO1 Vpu still reduced HLA-C protein levels at the cell surface (Fig. 2F), although it lost its ability to inhibit NF-κB (39). Together, these data demonstrate that NF-κB inhibition and HLA-C downmodulation are two distinct functions and suggest that HLA-C is targeted at the posttranscriptional level.

### Vpu does not decrease total cellular HLA-C levels.

**FIG 3:**
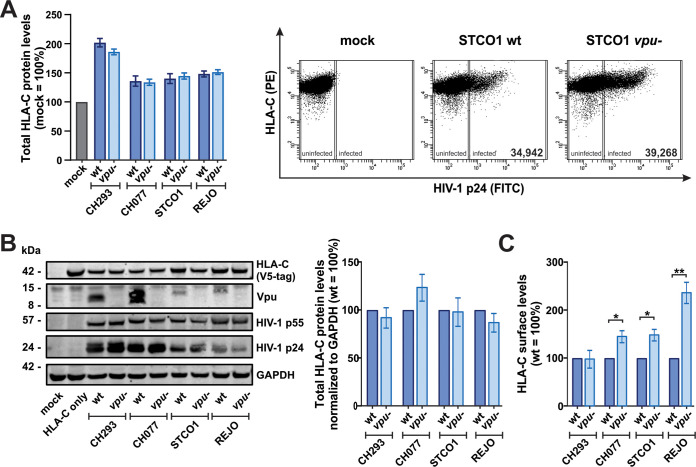
HIV-1 Vpu downmodulates HLA-C without changing total HLA-C protein levels. (A) PBMCs or purified CD4^+^ T cells were infected with the indicated wild-type (wt) or *vpu*-defective (*vpu−*) HIV-1 clones. At 3 days postinfection, total HLA-C protein levels were determined by permeabilization, staining, and subsequent flow cytometric analysis. Representative primary data are shown on the left. (B) HEK293T cells were cotransfected with the indicated molecular clones of wt or *vpu*-defective HIV-1 and an expression plasmid for V5-tagged HLA-C. All transfections except for wt and *vpu-*defective STCO were harvested at 2 days posttransfection for Western blot analyses. Cells transfected with wt and *vpu-*defective STCO were harvested at 3 days posttransfection to increase Vpu expression levels. One representative Western blot out of three is shown on the left. HLA-C protein levels were quantified and normalized to the GAPDH level. Wild-type-infected samples were set to 100% (right). (C) Cells were transfected as described above for panel B, and HLA-C protein levels at the cell surface were determined by flow cytometry at 2 days posttransfection. Bar diagrams show mean values from three to six independent experiments ± SEM. Asterisks indicate statistically significant differences (*, *P* < 0.05; **, *P* < 0.01).

Efficient HLA-C downmodulation depends on a phosphorylated DSGNES motif in the cytoplasmic domain of HIV-1 group M Vpu (2). This motif is also involved in the beta-transducin repeat-containing protein (β-TrCP)-dependent degradation of CD4 (43) and the adaptor protein (AP)/clathrin-dependent mistrafficking and subsequent degradation of tetherin (44). Nevertheless, the ultimate fate of HLA-C in the presence of Vpu has remained unclear. To investigate whether Vpu induces HLA-C degradation, we quantified total HLA-C levels in HIV-1-infected human peripheral blood mononuclear cells (PBMCs) and purified CD4^+^ T cells by flow cytometry. Our results showed that the levels do not differ between cells infected with the wild-type and *vpu* mutant viruses (Fig. 3A). Western blot analyses of transfected HEK293T cells confirmed that Vpu does not affect total HLA-C levels (Fig. 3B), although surface HLA-C levels were significantly reduced in this experimental setup (Fig. 3C), and these viruses were previously shown to counteract tetherin in a Vpu-dependent manner (39). This indicates that Vpu does not induce the degradation of HLA-C.

### The gain of antitetherin activity in HIV-1 group N may have come at the expense of HLA-C downmodulation.

**FIG 4:**
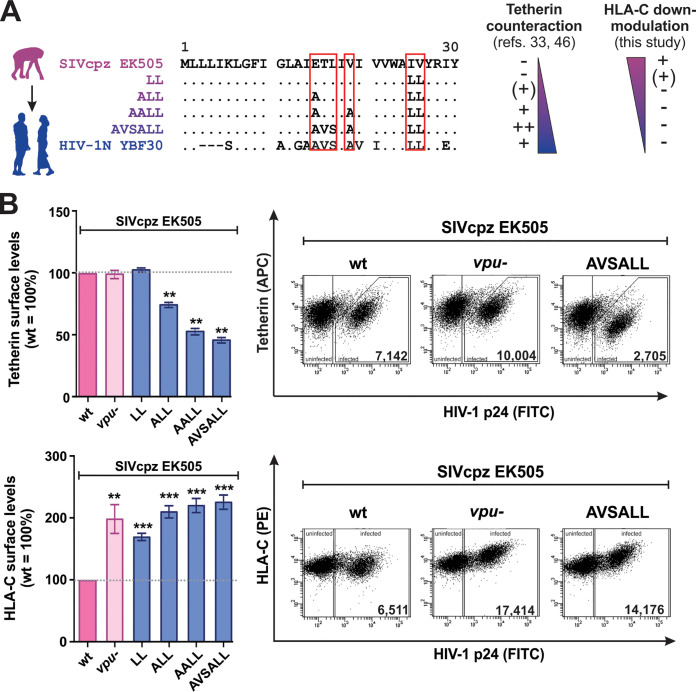
Gain of antitetherin activity may come at the cost of HLA-C downmodulation. (A) Alignment of the N termini of SIVcpz EK505 Vpu, HIV-1 N YBF30 Vpu, as well as several mutants mimicking adaptive changes upon cross-species transmission of SIVcpz to humans that ultimately resulted in the emergence of antitetherin activity (33, 46). (B) Purified CD4^+^ T cells were infected with SIVcpz EK505 carrying the indicated mutations in Vpu. At 3 days postinfection, surface levels of tetherin (top) and HLA-C (bottom) were determined by flow cytometry. (Left) Mean values from 3 to 11 independent experiments ± SEM. (Right) Representative primary data. Asterisks indicate statistically significant differences compared to the wild type (**, *P* < 0.01; ***, *P* < 0.001).

In addition to the DSGNES motif in the cytoplasmic domain, residues in the transmembrane domain of Vpu are involved in HLA-C downmodulation and tetherin counteraction (2, 3). This raised the possibility of an evolutionary conflict between these two Vpu functions. Upon cross-species transmission to humans, SIVcpz Vpu acquired several changes that ultimately resulted in the acquisition of antitetherin activity by HIV-1 groups M and N (33, 45). Notably, the changes acquired by HIV-1 group N Vpus only partly overlap those of group M Vpus (33). While most group M Vpus are able to perform both functions, we hypothesized that the gain of antitetherin activity may have contributed to the lack of HLA-C downmodulation activity in group N Vpus. To test this hypothesis, we took advantage of a set of Vpu mutants that were generated previously to mimic successive changes that may have occurred during the emergence of HIV-1 group N (33, 46) (Fig. 4A). In agreement with previous findings (33, 46), the exchange of 6 amino acids in the transmembrane domain (E15A, T16V, L17S, V19A, I25L, and V26L) was sufficient to confer efficient antitetherin activity to SIVcpz EK505 Vpu (Fig. 4B, top). However, the introduction of these same residues completely abrogated the ability of Vpu to decrease HLA-C levels on infected purified CD4^+^ T cells (Fig. 4B, bottom). Thus, HLA-C and the host restriction factor tetherin appear to have exerted opposing selection pressures on the transmembrane domain of HIV-1 group N Vpus, while most group M Vpus are able to perform both functions.

### HIV-2 uses Vif to downmodulate HLA-C.

**FIG 5:**
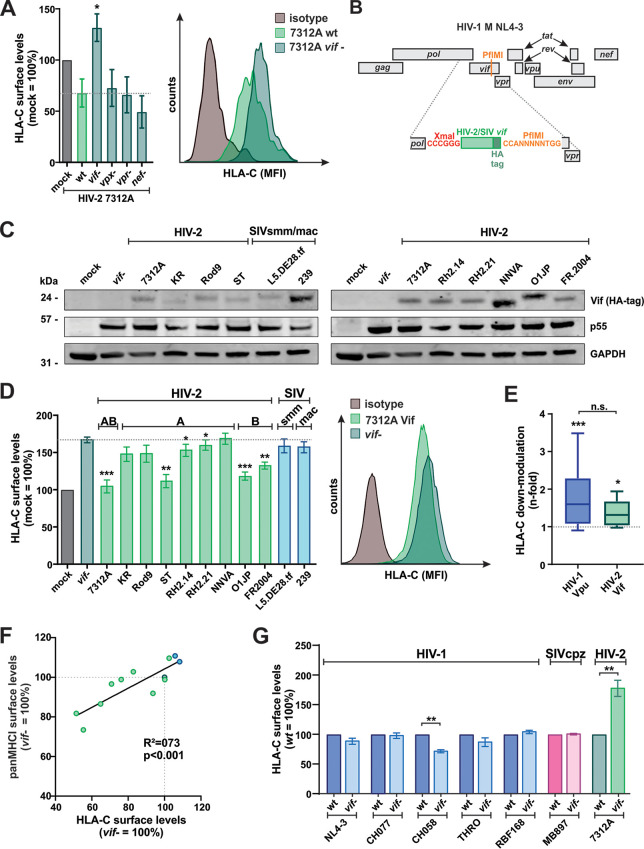
HIV-2 uses Vif to decrease HLA-C surface levels. (A) Purified CD4^+^ T cells were infected with the indicated wild-type or mutated variants of HIV-2 7312A coexpressing eGFP via an IRES. At 3 days postinfection, HLA-C levels at the surface of infected cells were determined by flow cytometry. Representative primary data are shown on the right. (B) Genome organization of the HIV-1 NL4-3 clone expressing heterologous HA-tagged Vifs (48). (C) PBMCs (left) or purified CD4^+^ T cells (right) were infected with the NL4-3 chimera shown in panel B expressing the indicated HIV-2 and SIVsmm/SIVmac Vifs. At 3 days postinfection, cells were harvested for Western blotting. (D) Purified CD4^+^ T cells were infected with NL4-3 chimeras expressing the indicated HIV-2 and SIVsmm/SIVmac Vifs. At 3 days postinfection, HLA-C surface levels were determined by flow cytometry. Representative primary data are shown on the right. (E) Average *n*-fold downmodulation of HLA-C by HIV-1 Vpus and HIV-2 Vifs. Values are derived from the experiments shown in Fig. 1 and in panel D. Box-and-whisker-plots illustrating the minimum, maximum, sample median, and first and third quartiles are shown. (F) PanMHC-I and HLA-C surface levels were determined as described above for panel D, and correlation analyses were performed. (G) Purified CD4^+^ T cells were infected with the indicated wild-type (wt) or *vif*-defective (*vif−*) lentiviral clones. At 3 days postinfection, HLA-C levels at the surfaces of infected cells were determined by flow cytometry. Bar diagrams show mean values from three to six independent experiments ± SEM. Asterisks indicate statistically significant differences compared to the wild type (A and G) or the *vif* stop mutant (D and E) (*, *P* < 0.05; **, *P* < 0.01; ***, *P* < 0.001).

In contrast to HIV-1, HIV-2 does not carry a *vpu* gene. Nevertheless, at least one HIV-2 clone (HIV-2 CRF01_AB 7312A) also decreases HLA-C levels in infected primary CD4^+^ T cells (2). This virus represents a recombinant form of HIV-2 groups A and B (47). To identify the viral protein(s) responsible for this activity, we infected purified CD4^+^ T cells with wild-type HIV-2 7312A as well as mutants lacking functional accessory genes. While the loss of Vpx, Vpr, or Nef expression had no significant effect on the HLA-C downmodulation activity of HIV-2 7312A, the loss of Vif disrupted this function (Fig. 5A). To investigate whether HLA-C downmodulation is a general feature of HIV-2 Vifs, we generated NL4-3 chimeras expressing different HIV-2 *vif* genes, including alleles of the most prevalent HIV-2 groups A and B. This particular construct (48) allows the exchange of *vif* genes without affecting the open reading frames of *pol* and *vpr* (Fig. 5B). Furthermore, NL4-3 Vpu expressed by this construct fails to downmodulate HLA-C (2) (Fig. 1C). Western blotting showed that all Vif proteins were expressed in primary human CD4^+^ T cells (Fig. 5C). As expected (Fig. 5A), wild-type 7312A Vif significantly decreased HLA-C surface levels when expressed from the HIV-1 NL4-3 backbone (Fig. 5D), which was also true for other HIV-2 Vifs (e.g., ST, O1JP, and FR2004). However, still other HIV-2 Vifs as well as select SIVsmm (representing the simian precursor of HIV-2) and related SIVmac Vifs failed to have an effect (Fig. 5D). This is similar to what has been observed for HLA-C modulation by different HIV-1 Vpus (Fig. 5E). HIV-2 Vifs reducing HLA-C surface levels also decreased the overall amount of all MHC class I molecules (panMHC-I) at the cell surface (Fig. 5F). Although HLA-C constitutes only a minor percentage of all MHC class I molecules on the cell surface (49), the amount of panMHC-I correlated well with that of HLA-C on infected cells expressing different Vifs (Fig. 5F). Importantly, we found no evidence for Vif-mediated HLA-C downmodulation in any HIV-1 or SIVcpz strains tested (Fig. 5G). Together, these findings demonstrate that different primate lentiviral lineages evolved alternative mechanisms to decrease HLA-C levels on infected cells.

### HLA-C modulation and APOBEC counteraction are separable functions of HIV-2 Vif.

**FIG 6:**
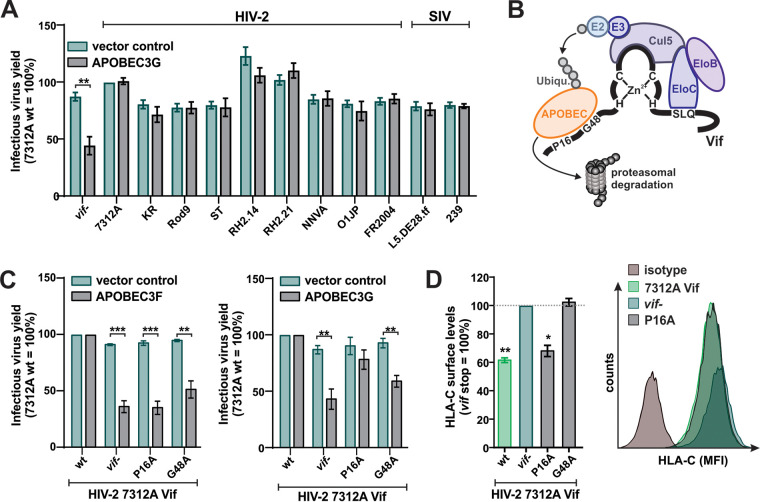
HLA-C downmodulation and APOBEC counteraction are separable HIV-2 Vif functions. (A) HEK293T cells were cotransfected with molecular clones of the indicated lentiviruses and an expression plasmid for APOBEC3G or the respective empty vector control. At 2 days posttransfection, the infectious virus yield was determined by infection of TZM-bl reporter cells. (B) Cartoon illustrating the recruitment of Cullin5 (Cul5) and Elongins B and C (EloB and EloC) by Vif that results in the ubiquitination and subsequent degradation of APOBECs. Vif residues mutated for the experiments in panels C and D and in Fig. 7C to E are indicated. (C) HEK293T cells were cotransfected with NL4-3 chimeras expressing the indicated variants of HIV-2 7312A Vif and an expression plasmid for APOBEC3F (left), APOBEC3G (right), or the respective empty vector control. At 2 days posttransfection, the infectious virus yield was determined by infection of TZM-bl reporter cells. (D) PBMCs were infected with NL4-3 chimeras expressing the indicated Vifs. At 3 days postinfection, surface HLA-C protein levels were determined by flow cytometry. Representative primary data are shown on the right. Bar diagrams show mean values from three to four independent experiments ± SEM. Asterisks indicate statistically significant differences (*, *P* < 0.05; **, *P* < 0.01; ***, *P* < 0.001).

The lentiviral accessory protein Vif is well known for its ability to induce the degradation of APOBEC proteins that would otherwise introduce lethal hypermutations in the viral genome (50). Intriguingly, APOBEC-induced mutations have been shown to affect the HLA-mediated presentation of viral antigens and CTL-mediated killing of infected cells (51–53). However, altered HLA-C surface expression in the presence of HIV-2 Vif is not merely a consequence of APOBEC counteraction, as all HIV-2 Vifs, including those that failed to downmodulate HLA-C (Fig. 5D), efficiently counteract APOBEC3G (Fig. 6A). This is in agreement with the finding that none of the HIV-1 Vifs tested decreases HLA-C surface levels (Fig. 5G), although several of them are known to counteract APOBEC3G (54). Thus, APOBEC3G counteraction and HLA-C downmodulation are genetically separable functions of HIV-2 Vif. Surprisingly, however, a single G48A point mutation in the N terminus of HIV-2 Vif that abrogates the counteraction of APOBEC3F and APOBEC3G (55) (Fig. 6B and C) also resulted in a complete loss of HLA-C downmodulation activity (Fig. 6D). In contrast, mutation P16A abrogated APOBEC3F counteraction (55) (Fig. 6C, left) but not HLA-C downmodulation by HIV-2 Vif (Fig. 6D).

### HIV-2 Vif-mediated HLA-C modulation requires Cullin5 and Elongin B/C binding motifs.

**FIG 7:**
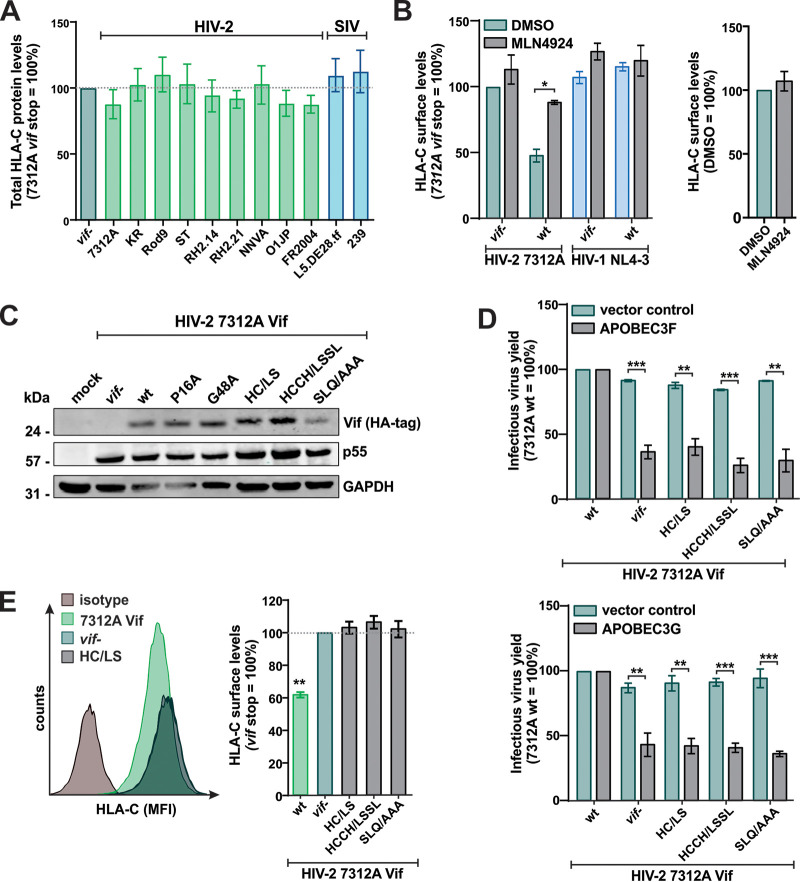
HIV-2 Vif-mediated HLA-C downmodulation requires Cullin5 and Elongin B/C binding motifs. (A) PBMCs were infected with NL4-3 chimeras expressing the indicated Vifs. At 3 days postinfection, total HLA-C protein levels were determined by flow cytometry. (B, left) Purified CD4^+^ T cells or PBMCs were infected with the indicated wild-type (wt) or *vif*-defective (*vif* stop, *vif*-) viruses. (Right) Mock-infected cells served as negative controls. At 3 days postinfection, cells were treated with the neddylation inhibitor MLN4924 or left untreated. HLA-C surface levels were determined by flow cytometry 24 h later. DMSO, dimethyl sulfoxide. (C) Purified CD4^+^ T cells were infected with NL4-3 chimeras expressing the indicated HIV-2 7312A Vifs. At 3 days postinfection, cells were harvested for Western blotting. One out of three independent Western blots is shown. (D) HEK293T cells were cotransfected with NL4-3 chimeras expressing the indicated variants of HIV-2 7312A Vif and an expression plasmid for APOBEC3F (top) or APOBEC3G (bottom) or the respective empty vector control. At 2 days posttransfection, the infectious virus yield was determined by infection of TZM-bl reporter cells. (E) Purified CD4^+^ T cells were infected with NL4-3 chimeras expressing the indicated 7312A Vif mutants. At 3 days postinfection, HLA-C surface levels were determined by flow cytometry. Representative primary data are shown on the left. Bar diagrams show mean values from three to four independent experiments ± SEM. Asterisks indicate statistically significant differences (*, *P* < 0.05; **, *P* < 0.01; ***, *P* < 0.001).

Lentiviral Vifs recruit the adapter proteins Elongin B and C (EloB/C) and the E3 ubiquitin ligase Cullin5 (Cul5) to mediate the ubiquitination and subsequent proteasomal degradation of APOBECs (56) (Fig. 6B). In contrast to APOBEC antagonism (57), however, the decrease of HLA-C surface expression by HIV-2 Vif is not associated with a significant decrease in total HLA-C protein levels in infected cells (Fig. 7A). It is well known that ubiquitination can modulate trafficking and surface levels of membrane proteins even in the absence of degradation (58). In line with a ubiquitin-dependent mechanism, HLA-C downmodulation by HIV-2 Vif was significantly impaired by the small-molecule inhibitor MLN4924 (Fig. 7B, left), which blocks the neddylation and, thus, the activation of the cullin-RING E3 ubiquitin ligase machinery (59, 60). HLA-C surface expression on mock-infected cells was not affected by MLN4924 (Fig. 7B, right).

To further investigate whether the recruitment of the E3 ubiquitin ligase complex is involved in HLA-C downmodulation, we generated mutants of HIV-2 7312A Vif lacking the Elongin C binding motif (SLQ147–149) (56) or a highly conserved HCCH motif (H110, C116, C135, and H141) that stabilizes the Vif-Cul5 interaction by complexing zinc (61, 62) (Fig. 6B). Western blot analyses showed that all Vif variants were efficiently expressed (Fig. 7C). As previously observed for HIV-1 Vifs (63, 64), the loss of the SLQ or HCCH motif abrogated the antagonization of APOBEC3F and APOBEC3G by HIV-2 7312A Vif (Fig. 7D). Furthermore, mutation of either of these motifs also resulted in a complete loss of HLA-C downmodulation activity (Fig. 7E). Thus, intact binding sites for Elongin B/C and Cullin5 as well as a glycine at position 48 are essential for Vif-mediated targeting of HLA-C. This demonstrates that APOBEC counteraction and HLA-C downmodulation are separable functions of HIV-2 Vif but depend on overlapping functional motifs.

### HIV-2 Vif-mediated HLA-C downmodulation sensitizes infected cells to NK cell killing.

**FIG 8:**
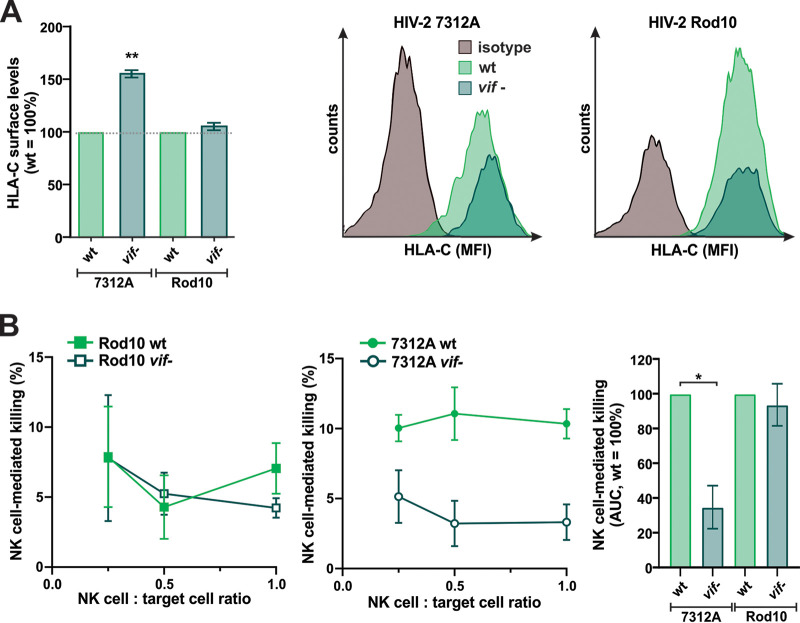
HIV-2 Vif-mediated HLA-C downmodulation sensitizes infected cells to NK cell killing. (A) Purified CD4^+^ T cells were infected with the indicated wild-type (wt) or *vif*-defective (*vif* stop) viruses. At 3 days postinfection, HLA-C surface levels were determined by flow cytometry. The bar diagram shows mean values from three independent experiments ± SEM. Representative fluorescence-activated cell sorter (FACS) data are shown on the right. (B) Purified CD4^+^ T cells infected with HIV-2 7312A or Rod10 (wild type or *vif* stop) were cocultured with autologous NK cells at three different ratios (4:1, 2:1, and 1:1). The killing of infected cells was monitored by flow cytometry. The right panel shows the mean areas under the curve (AUC) for the titration curves in the left two panels. Mean values from three independent experiments with three different donors ± SEM are shown. Asterisks indicate statistically significant differences (*, *P* < 0.05; **, *P* < 0.01).

Previous studies demonstrated that even modest changes in HLA-C surface levels affect the sensitivity of HIV-1-infected purified CD4^+^ T cells to NK cell- or CTL-mediated killing (2, 4). To investigate the functional consequences of Vif-mediated HLA-C downmodulation on the survival of HIV-2-infected cells, we cocultured infected primary CD4^+^ T cells with autologous NK cells and monitored cytotoxic killing. CD4^+^ T cells were infected with either HIV-2 7312A, which downmodulates HLA-C via Vif, or HIV-2 Rod10, which lacks this activity (Fig. 8A). Control experiments confirmed the expression of functional Vif proteins from both viruses since wild-type 7312A and Rod10, but not their *vif*-defective mutants, counteracted APOBEC3F and APOBEC3G (data not shown). At 2 days postinfection, CD4^+^ T cells and autologous NK cells were cocultured in three different ratios, and the percentage of killed cells was determined by flow cytometry 5 h later. In the case of Rod10, which does not downmodulate HLA-C, the presence of Vif had no significant effect on NK cell-mediated killing of infected cells (Fig. 8B, left). In contrast, the expression of Vif increased the killing of 7312A-infected cells under all three conditions tested (Fig. 8B, middle). Calculation of the area under the curve for independent experiments with three different donors confirmed that HIV-2 7312A Vif sensitizes infected cells to NK cell-mediated killing (Fig. 8B, right). These findings are consistent with increased missing-self detection upon HLA-C downmodulation (65) and demonstrate that even modest changes in HLA-C surface levels can have significant effects on NK cell-mediated killing of HIV-infected cells.

## DISCUSSION

Despite the key role of HLA-C in HIV-1 replication and pathogenesis (16, 66), its modulation by Vpu was not discovered until 2016 (2) and was analyzed only in the contexts of HIV-1 group M strains (2–6) and two HIV-2 clones (2). One reason for this late discovery was the reliance of previous studies on the T cell line-adapted HIV-1 clone NL4-3. In the present study, we analyzed 33 different Vpu proteins representing all four groups of HIV-1 as well as diverse SIV strains. This set included HIV-1 clones representing primary isolates from different stages of infection. We found that HLA-C downmodulation is not limited to HIV-1 group M but is also found in other groups of HIV-1 as well as SIVs infecting chimpanzees and different *Cercopithecus* monkeys. Analyzing different HIV-2 strains and mutants, a similar activity was found to be mediated by the HIV-2 Vif protein. Thus, the two human immunodeficiency viruses use different accessory proteins to evade HLA-C-mediated immune responses.

For both HIV-1 and HIV-2, the degree of HLA-C downmodulation varied substantially among different viral strains, ranging from 0% to 70%. This variation is similar to those observed in previous studies on HIV-1 group M Vpus (2–5). Characterization of almost 200 primary Vpus revealed that the degree of HLA-C downmodulation depends on the patient’s HLA-C genotype (3). On average, the efficiency of Vpu-mediated HLA-C downmodulation correlates with basal HLA-C expression levels in HIV-1-infected individuals (3), while a more recent study uncovered substantial variation even among Vpu alleles from the same subjects (4). The various degrees of conservation of HLA-C downmodulation among individual HIV-1 Vpu and HIV-2 Vif alleles may be explained by the dual role of HLA-C in retroviral replication. Although reduced HLA-C levels decrease antigen presentation and killing of infected cells by CTLs, a complete loss of HLA-C may not be beneficial for the virus. In a process of missing-self detection, NK cells are able to detect and kill cells lacking HLA-C on their cell surface (65). Consistent with this, we show that a reduction of HLA-C surface levels by HIV-2 Vif coincides with an increase in NK cell-mediated killing of HIV-2-infected CD4^+^ T cells. Thus, the HLA-C downmodulation activity of HIV-1 Vpu and HIV-2 may also depend on whether CTL- or NK cell-mediated immune responses prevail. The selection pressure exerted by NK cells is further reflected by the emergence of adaptive mutations in response to specific killer inhibitory receptors (KIRs) on NK cells, particularly in Vpu (67). Since not only HLA-C but also residual HLA-A and -B molecules are able to interact with inhibitory receptors on NK cells, optimal HLA-C downmodulation most likely also depends on the efficacy of Nef-mediated HLA-A and -B downmodulation. Finally, the widely varying HLA-C downmodulation capacity may also stem from the fact that both HIV-1 Vpu and HIV-2 Vif are multifunctional proteins whose activities may compete with each other. For example, increased tetherin expression negatively interferes with the ability of Vpu to downmodulate NTB-A and PVR (68). Similarly, we show here that HLA-C downmodulation may conflict with tetherin counteraction in HIV-1 group N strains as both functions depend on the same amino acid residues in the transmembrane domain of Vpu. Intriguingly, the amino acids that mediate antitetherin activity in group M Vpus only partially overlap those in group N Vpus (33). This may enable group M Vpus to downmodulate HLA-C and counteract tetherin at the same time. Group N Vpus may also be inactive because they are less stable than other Vpu proteins and frequently lack important functional domains (33), including the cytoplasmic DSGxxS motif, which recruits an E3 ubiquitin ligase complex that is required for full HLA-C downmodulation activity in HIV-1 group M strains (2). However, the apparent lack of HLA-C modulation by HIV-1 group N should be interpreted with caution since only two group N isolates were analyzed.

Bachtel and colleagues previously identified a 4-amino-acid motif in the transmembrane domain of human HLA-C that determines its interaction with HIV-1 Vpu (3). Consistent with this, residues 15, 16, 18, and 24 in the transmembrane of HIV-1 M Vpus are involved in HLA-C downmodulation (3). These residues partially overlap amino acids 15 to 19, 25, and 26 that we found to be required for HLA-C downmodulation by SIVcpz EK505 Vpu (33) (Fig. 4). The presence of HLA-C downmodulation activity in SIVcpz and HIV-1 groups M, O, and P together with the requirement of overlapping motifs suggest that this function evolved prior to the introduction of ape lentiviruses into humans. Similarly, Vpus from SIVgsn, SIVmus, and SIVden were also able to decrease HLA-C levels in infected cells, suggesting an ancient origin of this activity. This finding is surprising since HLA-C has been reported to be an evolutionarily rather young MHC-I molecule that can be found only in orangutans, chimpanzees, bonobos, gorillas, and humans but not in Old World monkeys such as the natural hosts of SIVgsn, SIVmus, or SIVden (69–72). This raises the possibility that the observed phenotype is not the result of a direct interaction of Vpu with HLA-C but involves a more indirect effect. Since HIV-1 and SIV Vpus are potent inhibitors of NF-κB activation (41), we hypothesized that reduced NF-κB-driven HLA-C transcription may contribute to decreased HLA-C surface levels. However, the HIV-1 and SIV Vpus tested did not affect *HLA-C* mRNA levels in infected cells, although they efficiently suppressed the NF-κB-mediated expression of interferon beta (IFN-β). Furthermore, Vpu mutants lacking the ability to suppress NF-κB activity revealed that this activity is dispensable for HLA-C downmodulation. If transcriptional regulation is not involved, why do SIVgsn, SIVmus, and SIVden Vpus downmodulate HLA-C? One possible explanation is that SIVgsn, SIVmus, and SIVden Vpus target a closely related MHC-I molecule in their natural hosts. Unfortunately, MHC-I sequences of the respective monkey hosts are not available (73). However, the transmembrane domains of Old World monkey MHC-B molecules share about 80% sequence identity with human HLA-C. In the study by Bachtel et al., a 4-amino-acid motif (LAVL) was identified to be required for the efficient interaction of HLA-C with HIV-1 group M Vpu (3). Duplications of this motif are regularly observed in MHC-B and MHC-C proteins from various primate species, including HLA-B, which is not targeted by Vpu (see Fig. S1 in the supplemental material). Thus, additional amino acid residues and/or the specific location of the LAVL motif in the transmembrane determines the sensitivity of HLA-C to Vpu. This raises the possibility that the observed downmodulation of human HLA-C by SIVgsn, SIVmus, and SIVden Vpus may be due to coincidental cross-reactivity with several MHC proteins. In line with a broader MHC-I downmodulation activity of lentiviral Vpus, two studies reported the downmodulation of HLA-G1 and (to a much lesser extent) HLA-E by HIV-1 Vpu (74, 75). These HLA molecules are expressed in *Cercopithecus* monkeys (69) but lack the LAVL motif (Fig. S1). Thus, future studies systematically analyzing the effects of diverse primate lentiviral Vpus on different MHC-I family members seem warranted to trace back the evolutionary origins of this immune evasion activity.

10.1128/mBio.00782-20.1 FIG S1 Protein sequence alignment of the transmembrane domains of representative primate MHC proteins. The previously described LAVL motif involved in HLA-C interactions (3) is highlighted in orange. Identical motifs elsewhere in the transmembrane domain are highlighted by red frames (HLA, human leukocyte antigen; Patr, Pan troglodytes [chimpanzee]; Papa, Pan paniscus [bonobo]; Gogo, Gorilla gorilla [Western gorilla]; Poab, Pongo abellii [Sumatran orangutan]; Mafa, Macaca fascicularis [crab-eating macaque]; Cemi, Cercopithecus mitis [blue monkey]; Ceat, Cercocebus atys [sooty mangabey]; Chsa, Chlorocebus sabaeus [green monkey]). Download FIG S1, TIF file, 0.8 MB. Copyright © 2020 Hopfensperger et al. 2020 Hopfensperger et al. This content is distributed under the terms of the Creative Commons Attribution 4.0 International license.

The selection advantage of HLA-C modulation by primate lentiviruses is corroborated by our finding that HIV-2 independently downmodulates HLA-C using its Vif protein. HLA-C downmodulations by HIV-2 Vif and HIV-1 Vpu share several common characteristics. In both cases, the degree of downmodulation varies considerably among different viral strains. Furthermore, HIV-2 Vif, like Vpu, decreases HLA-C surface levels without affecting *HLA-C* mRNA levels (data not shown). Mutational analyses revealed that binding sites for the Cul5 E3 ubiquitin ligase complex are required for HLA-C downmodulation. Furthermore, we found that inhibition of neddylation impairs Vif-mediated HLA-C downmodulation. These findings suggest that ubiquitination is involved in the modulation of HLA-C surface levels. Ubiquitination is a reversible modification that not only marks proteins for degradation but also regulates intracellular trafficking (58). The first experiments showed that HIV-2 Vif does not accelerate the endocytosis of surface HLA-C (data not shown). Future studies will reveal whether HIV-2 Vif interferes with the anterograde transport of HLA-C and/or modulates its recycling. Furthermore, it remains to be determined how HLA-C downmodulation by HIV-2 Vif interferes with its ability to counteract the host restriction factor APOBEC. Finally, it is tempting to speculate that Vif-mediated HLA-C downregulation evolved after zoonotic transmission of SIVsmm to humans since we found no evidence for this activity in SIVsmm or SIVmac and since sooty mangabeys lack a direct homolog of HLA-C. In such a scenario, HLA-C modulation by Vif would have evolved independently in HIV-2 groups A and B. However, only a single SIVsmm clone was analyzed in the present study, and a larger number of primary SIVsmm Vifs needs to be analyzed to test this hypothesis.

The identification of a novel HLA-C downmodulation mechanism in HIV-2 that evolved independently of Vpu-mediated HLA-C downmodulation in HIV-1 highlights the enormous plasticity of lentiviral accessory proteins. A similar example of convergent evolution was previously described for the inhibition of NF-κB activation. While HIV-1 and related lentiviruses use Vpu to suppress NF-κB-driven immune activation (37, 41), HIV-2 and other *vpu*-deficient viruses use their accessory protein Vpr, Vpx, or Nef to achieve this (76, 77). The evolution of independent evasion mechanisms in different primate lentiviral lineages illustrates the selection pressure exerted by the respective immune responses. Overall, our findings provide insights into HLA-C modulation during lentiviral infection that should be considered when developing therapeutic strategies based on CTL- or NK cell-mediated immune responses. This includes kick-and-kill approaches where CTLs and NK cells contribute to the elimination of HIV-1-infected cells.

## MATERIALS AND METHODS

### Cell culture.

Human embryonic kidney HEK293T cells (obtained from the American Type Culture Collection [ATCC]) were first described by DuBridge et al. (78). TZM-bl cells are a HeLa-derived reporter cell line and were obtained through the NIH AIDS Reagent Program, Division of AIDS, NIAID, NIH, from John C. Kappes, Xiaoyun Wu, and Tranzyme Inc. (79). HEK293T and TZM-bl cells were authenticated by the ATCC or the NIH and cultured in Dulbecco’s modified Eagle medium (DMEM) containing 10% heat-inactivated fetal calf serum (FCS) plus 2 mM glutamine, 100 μg/ml streptomycin, and 100 U/ml penicillin. All cell lines were tested for mycoplasma contamination every 3 months. Only mycoplasma-negative cells were used for this study.

Human PBMCs were isolated using a lymphocyte separation solution (Biocoll separating solution; Biochrom). In total, cells from 71 independent donations were used. CD4^+^ T cells were negatively isolated using the RosetteSep human CD4^+^ T cell enrichment cocktail (Stem Cell Technologies) according to the manufacturer’s instructions. Primary cells were cultured in RPMI 1640 medium containing 10% FCS, 2 mM glutamine, 100 μg/ml streptomycin, 100 U/ml penicillin, and 10 ng/ml interleukin 2 (IL-2) at 37°C in a 5% CO_2_ atmosphere. Before infection, cells were stimulated for 3 days with 1 μg/ml phytohemagglutinin (PHA). NK cells were negatively isolated from resting PBMCs using the NK cell enrichment cocktail (Stem Cell Technologies) according to the manufacturer’s instructions. NK cells were isolated and rested overnight in RPMI 1640 complete medium, without IL-2, on the day prior to the NK cell killing assay.

### APOBEC and HLA-C expression plasmids.

The pcDNA_APOBEC3G expression vector was kindly provided by Linda Chelico. To generate an expression plasmid for APOBEC3F, the respective open reading frame was inserted via XbaI/MluI into a pCG vector coexpressing blue fluorescent protein (BFP) via an internal ribosome entry site (IRES) (primers A3F_fw [CGTCTAGAATATGAAGCCTCACTTCAG] and A3F_rev [CTACGCGTTCACTCGAGA ATCTCCTGC]). The V5-tagged HLA-C expression plasmid was described previously (80).

### Infectious molecular clones of HIV-1, HIV-2, and SIV.

Infectious molecular clones of HIV-1 group M CH293 (81), STCO1 (81), CH077 (82), REJO (82), CH058 (82), and AD8 (83); HIV-1 group N DJO0131 (33); HIV-1 group O CMO2.5 (84) and RBF206 (85); as well as SIVcpz MB897 (86), EK505 (86), and GAB1 (87) were described previously. The infectious molecular clone of HIV-1 group P RBF168 was generated from a plasma viral isolate obtained 4 years after diagnosis by coculture with donor PBMCs (88, 89). Viral RNA was extracted from the culture supernatant and subjected to cDNA synthesis. Single-genome sequencing of 3′ and 5′ half-genomes was then performed to generate an isolate consensus sequence. The absence of ambiguous bases in this consensus indicated a limiting-dilution-derived isolate. Four overlapping fragments were chemically synthesized and assembled to generate an infectious molecular clone (GenBank accession number KY953207).

HIV-2 7312A coexpressing enhanced green fluorescent protein (eGFP) via an IRES element was generated by overlap extension PCR. Briefly, the 3′ long terminal repeat (LTR) fragment overlapping *nef* was PCR amplified with Phire Hot Start II DNA polymerase (Thermo Scientific), and a multiple-cloning site (MCS) containing the restriction sites AscI and SalI was added to the 5′ end. This PCR fragment was introduced after the stop codon of HIV-2 7312A *nef* by overlap extension PCR using the single-cutter restriction sites HindIII in *env* and KpnI downstream of the 3′ LTR. Afterward, the IRES-*eGFP* cassette was PCR amplified with the flanking restriction sites AscI and SalI from the previously described pBR_NL4-3 IRES eGFP construct (90) and inserted into the cloned HIV-2 7312A variant harboring the AscI/SalI cloning site. The single restriction site KpnI downstream of the 3′ LTR was changed to MluI using the QuikChange II XL site-directed mutagenesis kit (Agilent). Finally, the pBluescript II KS(+) backbone was replaced by the pBR322 backbone by the use of the flanking restriction sites MluI and NotI.

Premature stop codons and other point mutations in *vpu*, *vif*, *vpx*, and *vpr* were introduced using overlap extension PCR, a QuikChange II XL site-directed mutagenesis kit (Agilent), or a Q5 site-directed mutagenesis kit (New England BioLabs). SIVcpz EK505 *vpu*-deficient and AVSALL mutants were described previously (46). The respective LL, ALL, and AALL mutants were generated via overlap extension PCR and inserted into the proviral backbone via SbfI/BlpI. Variants of HIV-1 NL4-3 expressing different *vpu* alleles were described previously (32). Briefly, the overlap of *vpu* and *env* was eliminated, and heterologous *vpu* alleles were inserted via SacII/NcoI. Env expression was restored by inserting an IRES upstream of *env* via NcoI. HIV-1 NL4-3 clones encoding C-terminally hemagglutinin (HA)-tagged Vifs are based on HIV-1^VS^ described previously by Stern and colleagues (48). In this proviral construct, the *vif* open reading frame was exchanged via XmaI/EcoRI without altering integrase or Vpr protein sequences. A variant harboring 7312A *vif* with two premature stop codons served as a negative control. The respective 7312A constructs harboring P16A, G48A, HC/LS, HCCH/LSSL, or SLQ/AAA *vif* mutations were generated using overlap extension PCR and inserted into the NL4-3 backbone via XmaI/EcoRI.

### Transfection, generation of virus stocks, and infectious virus release assay.

HEK293T cells were transfected using a standard calcium phosphate method. To generate virus stocks, HEK293T cells were seeded in 6-well plates and transfected with 5 μg proviral DNA. For pseudotyping, cells were cotransfected with 4 μg proviral DNA and 1 μg of an expression plasmid for the vesicular stomatitis virus glycoprotein (VSV-G) (pHIT-G_VSV-G) (91). At 2 days posttransfection, cell culture supernatants were harvested and cleared of cell debris by centrifugation at 1,700 × *g* for 4 min. For infection of primary CD4^+^ T cells used for qRT-PCR, virus stocks were concentrated 20-fold via ultracentrifugation (96,325 × *g* for 120 min) to achieve higher infection rates. For all other infections, virus stocks were not concentrated.

To assess the ability of Vif to counteract APOBEC3G or APOBEC3F, HEK293T cells were seeded in 6-well plates and transfected with 4 μg of the proviral construct in the presence of either 1 μg of the APOBEC3G, 0.25 μg of the APOBEC3F expression plasmid, or the corresponding empty vector. At 40 h posttransfection, the infectious virus yield was determined by infecting TZM-bl reporter cells.

### Infection of TZM-bl reporter cells.

To determine the infectious virus yield, 6,000 TZM-bl cells were seeded in 96-well plates and infected with 2 to 50 μl of the cell culture supernatant in triplicate on the following day. At 3 days postinfection, β-galactosidase reporter gene expression was determined using the GalScreen kit (Applied Bioscience) according to the manufacturer’s instructions.

### Infection of PBMCs and purified CD4^+^ T cells.

To determine Vpu-mediated effects on HLA-C, CD4, panMHC-I, and tetherin surface levels, 1 million to 1.5 million activated PBMCs or purified CD4^+^ T cells were infected in 96-well plates with 120 μl nonconcentrated VSV-G-pseudotyped HIV-1 via spinoculation (1,200 × *g* for 120 min at 37°C). Afterward, cells were cultured in 2 ml supplemented RPMI medium in 6-well plates. For qRT-PCR, purified CD4^+^ T cells were infected in the same way using 20-fold-concentrated virus stocks. Here, wild-type and mutant viruses of each virus strain were adjusted for infectivity using a TZM-bl reporter cell assay.

### HLA promoter reporter assay.

To determine the NF-κB responsiveness of HLA promoters, we generated reporter constructs expressing firefly luciferase under the control of the HLA-B or HLA-C promoter. HLA-B and HLA-C promoter sequences were amplified from genomic DNA (gDNA) of mucous membrane cells obtained by buccal swabs (primer HLA-B/C_fw CCAGGAGGAGAAGTGAAGGGG]) and inserted into pGL3-enhancer (Promega) via KpnI/XhoI. A previously described NF-κB reporter vector served as a control (76). HEK293T cells were cotransfected with 0.2 μg of the firefly luciferase reporter construct and 0.1 μg of a *Gaussia* luciferase vector under the control of the pTAL promoter for normalization. All transfections were performed in 96-well plates, in triplicates, using the calcium phosphate method. NF-κB signaling was activated by the cotransfection of a constitutively active mutant of IκB kinase β (IKKβ) (0.8 μg). At 40 h posttransfection, a dual-luciferase assay was performed, and the firefly luciferase signals were normalized to the corresponding *Gaussia* luciferase control values.

### Western blotting.

To determine cellular and viral protein expression, infected purified CD4^+^ T cells or PBMCs were washed in phosphate-buffered saline (PBS), lysed in Western blot lysis buffer (150 mM NaCl, 50 mM HEPES, 5 mM EDTA, 0.1% NP-40, 500 mM Na_3_VO_4_, 500 mM NaF [pH 7.5]), and cleared by centrifugation at 20,800 × *g* for 20 min at 4°C. Lysates were mixed with protein sample loading buffer supplemented with 10% β-mercaptoethanol and heated at 95°C for 5 min. Proteins were separated on NuPAGE 4% to 12% Bis-Tris gels, blotted onto Immobilon-FL polyvinylidene difluoride (PVDF) membranes, and stained using primary antibodies (Abs) directed against HA (catalog number ab18181; Abcam), V5 (catalog number ab206571; Abcam), HIV-1 p24 (catalog number ab9071; Abcam), and glyceraldehyde-3-phosphate dehydrogenase (GAPDH) (catalog number 607902; BioLegend); Vpu subtype C antiserum (catalog number 11942; NIH Reagent Program); polyclonal rabbit anti-HIV-1 Vpu Ab 32-81 (kindly provided by Ulrich Schubert); and infrared dye-labeled secondary antibodies (IRDye; Li-Cor). A signal enhancer Hikari kit for Western blotting and enzyme-linked immunosorbent assays (ELISAs) (Nacalai Tesque) was used for Vpu and p24 staining. Proteins were detected using a Li-Cor Odyssey scanner, and band intensities were quantified using Li-Cor Image Studio Lite version 3.1.

### Flow cytometry.

To determine the purity of CD4^+^ T cells after isolation, cells were simultaneously stained for surface CD4 (peridinin chlorophyll protein [PerCP], catalog number 550631; BD Pharmingen) and CD11c (fluorescein isothiocyanate [FITC], catalog number ab22540; Abcam). To monitor HLA-C, MHC class I, or tetherin levels, infected PBMCs or purified CD4^+^ T cells were analyzed by flow cytometry essentially as described previously (2–4). At 3 days postinfection, cells were stained for the surface expression of HLA-C (clone DT9) (phycoerythrin [PE] conjugated [catalog number 566372; Becton, Dickinson] or unconjugated [catalog number MABF233; Merck Millipore] in combination with Alexa Fluor 647 secondary antibody [catalog number A-31571; Thermo Fisher]), MHC class I (clone G46-2.6) (PE, catalog number 555553; Becton, Dickinson), or tetherin (allophycocyanin [APC], catalog number 348410; BioLegend) using the Fix&Perm cell fixation and permeabilization kit (Nordic MUbio) according to the manufacturer’s instructions. HIV-1- and HIV-2-infected cells were identified by intracellular staining for p24 and p27, respectively (FITC, catalog number 6604665; Beckman Coulter), or proviral eGFP expression. In some experiments, cells were additionally stained for CD4 (APC, from Thermo Fisher Scientific [catalog number MHC0405] or Becton, Dickinson [catalog number 555349]) to distinguish productively (i.e., p24/27-positive [p24/27^+^] and CD4-negative [CD4^−^]) from nonproductively (i.e., p24/27-positive and CD4-positive) infected cells. Isotype control antibodies (HLA-C antibodies from Becton, Dickinson [catalog number 555058], or Merck Millipore [catalog number MABC006]; MHC-I antibody from Becton, Dickinson [catalog number 555749]; tetherin antibody from BD Pharmingen [catalog number 555751]; and CD4 antibody from Thermo Fisher Scientific [catalog number MG2A05]) were used to determine background fluorescence and unspecific antibody binding. The mean fluorescence intensity (MFI) ratios of infected and uninfected cells were calculated to determine changes in protein surface levels of HLA-C. Flow cytometric analyses were performed on a BD Canto II flow cytometer using BD FACSDiva software (BD Biosciences). Histogram overlays were generated using FlowJo v10.6 (Becton, Dickinson).

### Inhibitor treatments.

PBMCs were infected with the indicated wt or *vif*-deficient virus stocks via spinoculation as described above. At 3 days postinfection, cells were treated with 500 nM the NEDD8-activating enzyme inhibitor MLN4924 (MedChemExpress) for 24 h. Subsequently, HLA-C surface expression was determined via flow cytometry as described above.

### qRT-PCR.

Total RNA was isolated and purified from pelleted infected CD4^+^ T cells using the RNeasy Plus minikit (Qiagen) according to the manufacturer’s instructions. Cells were homogenized by vortexing for 30 s. Subsequent gDNA digestion was performed using the DNA-free DNA removal kit (Thermo Fisher Scientific) if necessary. The maximal amount of RNA was reverse transcribed with the PrimeScript RT reagent kit (Perfect real time) (TaKaRa) using oligo(dT) primers and random hexamers. cDNA was subjected to qRT-PCR using primer/probe sets for human HLA-C, *IFNB1*, and GAPDH (Thermo Fisher Scientific). HLA-C and GAPDH were run in a duplex format. *IFNB1* and GAPDH were run as singleplex reactions. Samples were analyzed in triplicates. Threshold cycle (*C_T_*) data were processed relative to the GAPDH control.

### NK cell assay.

At 2 days postinfection, infected primary CD4^+^ T cells were stained with a viability dye (AquaVivid; Thermo Fisher Scientific) and a cell proliferation dye (eFluor670; eBioscience) and used as target cells. Unstimulated autologous purified NK cells, stained with another cellular marker (eFluor450 cell proliferation dye; eBioscience), were counted and added at different NK-to-target cell ratios in 96-well V-bottom plates (Corning) as previously described (68). The plates were subsequently centrifuged for 1 min at 300 × *g* and incubated at 37°C with 5% CO_2_ for 5 h. Productively infected cells (CD4^−^ p24^+^ cells) were then identified by cell surface staining for CD4 (FITC, clone OKT4; BioLegend), followed by intracellular staining for p24 (PE, clone KC57; Beckman Coulter). Samples were acquired on an LSRII cytometer (BD Biosciences), and data analysis was performed using FlowJo vX.0.7 (Becton, Dickinson). The percentage of direct killing was calculated with the formula (% of CD4^−^ p24^+^ cells in targets) − (% of CD4^−^ p24^+^ cells in targets plus NK cells)/(% of CD4^−^ p24^+^ cells in targets) by gating on live target cells.

### Statistical analyses.

All statistical calculations were performed with a two-tailed unpaired Student *t* test or a one-sample *t* test using GraphPad Prism 7. *P* values of ≤0.05 were considered significant.

### Sequence analyses.

Vpu and MHC amino acid sequences were aligned using MultAlin (92).

### Ethics statement.

Experiments involving human peripheral blood mononuclear cells were reviewed and approved by the Institutional Review Board (i.e., the Ethics Committee of Ulm University), and individuals and/or their legal guardians provided written informed consent prior to donating blood. All blood samples were anonymized before use.
